# Continuous Writing, Reviewing, and Editing by Physicians

**DOI:** 10.31662/jmaj.2024-0017

**Published:** 2024-08-09

**Authors:** Vitorino Modesto dos Santos

**Affiliations:** 1 Armed Forces Hospital, Catholic University of Brasília-DF, Taguatinga, Brazil

**Keywords:** Case report, letters, medical education, undergraduate, postgraduate

## Dear Editor,

I read interesting articles about writing, reviewing, and editing medical manuscripts published in this Journal by Matsubara and Saeki S ^(1), (2), (3)^. They specifically focused on the panorama in Japan, but they should be an example to be solved by educators from other countries. The analysis of the cornerstone items related to the early adequate engagement of medical students in the production of scientific articles gave rise to a very favorable impression. Indeed, medical writing technique should be included in the curriculum of medical students to develop their capacity to prepare manuscripts, firstly letters to the Editor and case reports ^(1), (2), (3)^. Therefore, senior preceptors should also offer to young students the available time and necessary material resources to write, besides providing their support with analysis and edifying guidelines that can result in greater incentive to continue with such important desideratum ^(1), (2), (3)^. In addition to increasing their interest in relation to anamnesis and physical examination data, laboratory determinations, imaging, and anatomopathological findings ^(4), (5)^, this really can favor the strong basis of the future doctor-patient relationship from the first periods of medical course. Another cornerstone item of manuscript preparation should be continuity, in spite of difficulties faced in the initial steps, because one really learns to write while persisting writing ^(2)^. Furthermore, this promising engagement in the preparation of articles will facilitate the further presentation of scientific works at medical conferences and future research development. Notably, the contingent of new producers of scientific articles will increase, resulting in the reduction of under-reported cases, in addition to the under-diagnosis and misdiagnosis rate ^(1), (4)^.

In this context, it seems useful to add some short comments on two Brazilian articles that highlighted major causes and consequences of “hyposkillia” that may occur in undergraduate and postgraduate education programs in different regions of the whole world ^(4), (5)^. A very important activity for future doctors to gain more solid knowledge about the illnesses that victimized their patients is represented by the routine of clinic-pathological sessions, with the participation of specialists from different areas, and led by the most experienced advisors ^(5)^. Besides, the use of clinical and complementary findings, including conclusive diagnostic images, constitute one of the most consistent ways to elaborate the manuscripts to be published. Brazilian study groups include medical students from the initial periods of the course working with the orientation of senior preceptors and professors with MSc and PhD degrees ^(4)^.

## Article Information

### Conflicts of Interest

None

### Author Contributions

VMS wrote the first manuscript, edited and approved the final manuscript.

### ORCID iD

Vitorino Modesto dos Santos: https://orcid.org/0000-0002-7033-6074

### Approval by Institutional Review Board (IRB)

IRB approval was not required for this study.

### Informed Consent

Informed consent was not required for this study.
